# (4-Hydroxy­methyl-1*H*-imidazole-κ*N* ^3^)bis­(tri-*tert*-butoxy­silanethiol­ato-κ^2^ *O*,*S*)cadmium(II)

**DOI:** 10.1107/S1600536808035642

**Published:** 2008-11-08

**Authors:** Anna Dołęga, Katarzyna Baranowska, Żaneta Jarząbek

**Affiliations:** a Department of Inorganic Chemistry, Faculty of Chemistry, Gdańsk University of Technology, 11/12 G. Narutowicz St., 80952 PL Gdańsk, Poland

## Abstract

The Cd^II^ atom in the title compound, [Cd(C_12_H_27_O_3_SSi)_2_(C_4_H_6_N_2_O)], is penta­coordinated by two O and two S atoms from the *O*,*S*-chelating silanethiol­ate residue and one N from the 4-hydroxy­methyl­imidazole ligand and shows a strongly distorted trigonal-bipyramidal geometry. The title complex is isostructural with its zinc analog. The hydroxy group of the ligand is involved in intra­molecular O—H⋯S hydrogen bonding and also acts as an acceptor in the formation of an inter­molecular N—H⋯O hydrogen bond, which links mol­ecules of the complex into zigzag chains parallel to the *b* axis. One of the *tert*-butyl groups is disordered over two orientations with occupancies of 0.557 (12):0.443 (12).

## Related literature

For similar compounds, see: Dołęga *et al.* (2006 ▶, 2007 ▶, 2008 ▶). For the synthetic procedure, see: Wojnowski *et al.* (1992 ▶).
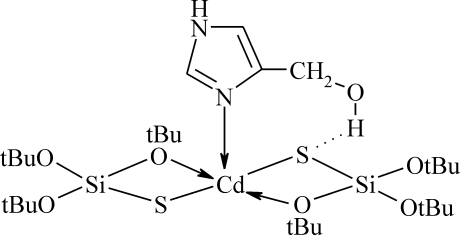


## Experimental

### 

#### Crystal data

[Cd(C_12_H_27_O_3_SSi)_2_(C_4_H_6_N_2_O)]
*M* *_r_* = 769.48
Monoclinic, 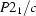

*a* = 16.3362 (4) Å
*b* = 9.1279 (2) Å
*c* = 26.6535 (6) Å
β = 92.258 (2)°
*V* = 3971.36 (16) Å^3^
*Z* = 4
Mo *K*α radiation
μ = 0.75 mm^−1^
*T* = 120 (2) K
0.13 × 0.10 × 0.06 mm

#### Data collection

Oxford Diffraction KM-4-CCD diffractometer
Absorption correction: analytical (*CrysAlis RED*; Oxford Diffraction, 2006 ▶) *T*_min_ = 0.856, *T*_max_ = 0.923
27726 measured reflections
7385 independent reflections
6448 reflections with *I* > 2σ(*I*)
*R*_int_ = 0.026

#### Refinement

*R*[*F* ^2^ > 2σ(*F* ^2^)] = 0.032
*wR*(*F* ^2^) = 0.096
*S* = 1.14
7385 reflections
376 parameters
H atoms treated by a mixture of independent and constrained refinement
Δρ_max_ = 1.14 e Å^−3^
Δρ_min_ = −0.80 e Å^−3^

### 

Data collection: *CrysAlis CCD* (Oxford Diffraction, 2006 ▶); cell refinement: *CrysAlis RED* (Oxford Diffraction, 2006 ▶); data reduction: *CrysAlis RED*; program(s) used to solve structure: *SHELXS97* (Sheldrick, 2008 ▶); program(s) used to refine structure: *SHELXL97* (Sheldrick, 2008 ▶); molecular graphics: *ORTEP-3 for Windows* (Farrugia, 1997 ▶); software used to prepare material for publication: *WinGX* (Farrugia, 1999 ▶).

## Supplementary Material

Crystal structure: contains datablocks I, global. DOI: 10.1107/S1600536808035642/gk2173sup1.cif

Structure factors: contains datablocks I. DOI: 10.1107/S1600536808035642/gk2173Isup2.hkl

Additional supplementary materials:  crystallographic information; 3D view; checkCIF report

## Figures and Tables

**Table:** 

Cd1—N1	2.2653 (19)
Cd1—S1	2.4599 (6)
Cd1—S2	2.4633 (6)
Cd1—O1	2.5511 (16)
Cd1—O4	2.5516 (16)
S1—Si1	2.1047 (8)
S2—Si2	2.0872 (8)

**Table:** 

N1—Cd1—S1	110.88 (5)
S1—Cd1—S2	144.16 (2)
S1—Cd1—O1	71.84 (4)
O1—Cd1—O4	176.43 (5)

**Table 2:** Hydrogen-bond geometry (Å, °)

*D*—H⋯*A*	*D*—H	H⋯*A*	*D*⋯*A*	*D*—H⋯*A*
N2—H2⋯O7^i^	0.88	1.96	2.759 (3)	151
O7—H7*D*⋯S1	0.81 (3)	2.41 (3)	3.2119 (19)	176 (3)

Symmetry code: (i) 
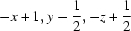
.

**Fig. 1.**
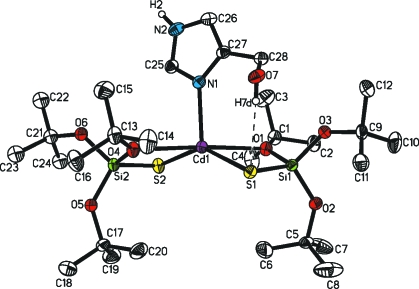
A view of the title molecule, showing the atom-labelling scheme. Displacement ellipsoids are drawn at the 50% probability level and H atoms, except those from OH and NH groups, have been omitted. Hydrogen bonds are indicated with dashed lines. Only the major conformation for the disordered methyl groups (C24-C25) is shown.

**Fig. 2.**
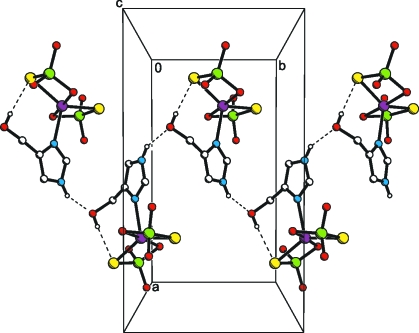
A view of one-dimensional chains of molecules linked by hydrogen bonds along the c axis. All tBu groups have been omitted for clarity. Hydrogen bonds are indicated with dashed lines.

## supplementary crystallographic information

### Comment

We investigate structural and spectroscopic features of simple inorganic complexes - models of the catalytic site of the enzyme alcohol dehydrogenase. We use tri-*tert*- butoxysilanethiol as a source of thiolate function and substituted imidazoles as histidine analogs (Dołęga *et al.*, 2008, and references therein)

Complex (I) is isostructural with (4-hydroxymethyl-1*H*- imidazole-κ*N*)bis(tri-*tert*-butoxysilanethiolatoκ^2^:*O*,*S*)zinc(II) (Dołęga *et al.*, 2008) thus the overall geometry, crystal packing and hydrogen bonds parameters in the title compound and its zinc analog are practically the same. The differences in metal-ligand bond lengths stem from the differences in zinc and cadmium radii. Additionally, the title complex shows a very large (> 140°) S—Cd—S angle, considerably larger than the respective angle in the zinc analog, a feature very characteristic of five-coordinated cadmium tri-*tert*-butoxysilanethiolates with CdNO_2_S_2_ kernels (Dołęga *et al.*, 2006, Dołęga *et al.*, 2007).

The molecular structure of (I) is shown in Fig. 1 and crystal packing diagram is presented in Fig.2.

### Experimental

The title compound was prepared and crystallized by slow evaporation of solvent from toluene solution containing equimolar amounts of cadmium bis(tri-*tert*-butoxysilanethiolate) (Wojnowski *et al.* 1992) and 4(5)-hydroxymethylimidazole.

### Refinement

All C–H hydrogen atoms were refined as riding on carbon atoms with methyl C–H = 0.98 Å, methylene C–H = 0.99 Å, aromatic C–H = 0.95 Å and *U*_iso_(H)=1.2 *U*_eq_(C) for aromatic and methylene CH and 1.5*U*_eq_(C) for methyl groups. H7D (OH) was found in the difference Fourier map and refined without constraints. Atoms C22—C24 are disordered over two positions (0.557 (12)/0.443 (12)). All atoms in the disordered *tert*-butyl group were set as isotropic (C21—C24). The voids of 61 Å^3^, present in the structure, are surrounded by *tert*-butyl groups and contain only insignificat residual electron density (the highest residual density in the void is 0.70 e/A^3^).

### Crystal data

**Table:**

[Cd(C_12_H_27_O_3_SSi)_2_(C_4_H_6_N_2_O)]	*F*_000_ = 1624
*M**_r_* = 769.48	*D*_x_ = 1.287 Mg m^−^^3^
Monoclinic, *P*2_1_/*c*	Mo *K*α radiation λ = 0.71073 Å
Hall symbol: -P 2ybc	Cell parameters from 25578 reflections
*a* = 16.3362 (4) Å	θ = 2.0–32.5º
*b* = 9.1279 (2) Å	µ = 0.75 mm^−^^1^
*c* = 26.6535 (6) Å	*T* = 120 (2) K
β = 92.258 (2)º	Prism, colourless
*V* = 3971.36 (16) Å^3^	0.13 × 0.10 × 0.06 mm
*Z* = 4	

### Data collection

**Table:**

Oxford Diffraction KM-4-CCD diffractometer	7385 independent reflections
Monochromator: graphite	6448 reflections with *I* > 2σ(*I*)
Detector resolution: 8.1883 pixels mm^-1^	*R*_int_ = 0.026
*T* = 120(2) K	θ_max_ = 25.5º
0.6° wide ω scans	θ_min_ = 2.0º
Absorption correction: analytical(CrysAlis RED; Oxford Diffraction, 2006)	*h* = −19→19
*T*_min_ = 0.856, *T*_max_ = 0.923	*k* = −11→11
27726 measured reflections	*l* = −32→31

### Refinement

**Table:**

Refinement on *F*^2^	Secondary atom site location: difference Fourier map
Least-squares matrix: full	Hydrogen site location: inferred from neighbouring sites
*R*[*F*^2^ > 2σ(*F*^2^)] = 0.032	H atoms treated by a mixture of independent and constrained refinement
*wR*(*F*^2^) = 0.096	*w* = 1/[σ^2^(*F*_o_^2^) + (0.067*P*)^2^] where *P* = (*F*_o_^2^ + 2*F*_c_^2^)/3
*S* = 1.14	(Δ/σ)_max_ = 0.001
7385 reflections	Δρ_max_ = 1.14 e Å^−^^3^
376 parameters	Δρ_min_ = −0.80 e Å^−^^3^
Primary atom site location: structure-invariant direct methods	Extinction correction: none

### Special details

**Table:**

Geometry. All e.s.d.'s (except the e.s.d. in the dihedral angle between two l.s. planes) are estimated using the full covariance matrix. The cell e.s.d.'s are taken into account individually in the estimation of e.s.d.'s in distances, angles and torsion angles; correlations between e.s.d.'s in cell parameters are only used when they are defined by crystal symmetry. An approximate (isotropic) treatment of cell e.s.d.'s is used for estimating e.s.d.'s involving l.s. planes.
Refinement. Refinement of *F*^2^ against ALL reflections. The weighted *R*-factor *wR* and goodness of fit *S* are based on *F*^2^, conventional *R*-factors *R* are based on *F*, with *F* set to zero for negative *F*^2^. The threshold expression of *F*^2^ > σ(*F*^2^) is used only for calculating *R*-factors(gt) *etc*. and is not relevant to the choice of reflections for refinement. *R*-factors based on *F*^2^ are statistically about twice as large as those based on *F*, and *R*- factors based on ALL data will be even larger.

### Fractional atomic coordinates and isotropic or equivalent isotropic displacement parameters (Å^2^)

**Table:**

	*x*	*y*	*z*	*U*_iso_*/*U*_eq_	Occ. (<1)
C1	0.26185 (15)	0.7084 (3)	0.33154 (9)	0.0242 (5)	
C2	0.22265 (16)	0.6933 (3)	0.38215 (9)	0.0295 (6)	
H2A	0.163	0.687	0.3771	0.044*	
H2B	0.2366	0.7788	0.403	0.044*	
H2C	0.2431	0.6043	0.399	0.044*	
C3	0.35460 (16)	0.7180 (3)	0.33821 (12)	0.0421 (7)	
H3A	0.3692	0.7997	0.3607	0.063*	
H3B	0.3784	0.734	0.3055	0.063*	
H3C	0.3759	0.6264	0.3528	0.063*	
C4	0.2371 (2)	0.5823 (3)	0.29714 (12)	0.0403 (7)	
H4A	0.1772	0.5777	0.2935	0.06*	
H4B	0.258	0.4902	0.3115	0.06*	
H4C	0.26	0.5975	0.2641	0.06*	
C5	0.01249 (14)	0.8699 (3)	0.30993 (9)	0.0238 (5)	
C6	0.0257 (2)	0.8463 (5)	0.25496 (12)	0.0602 (11)	
H6A	0.0709	0.777	0.2511	0.09*	
H6B	0.0394	0.9399	0.2393	0.09*	
H6C	−0.0243	0.8067	0.2387	0.09*	
C7	−0.0044 (2)	0.7254 (4)	0.33537 (13)	0.0545 (10)	
H7A	0.0412	0.6579	0.3303	0.082*	
H7B	−0.0551	0.683	0.3209	0.082*	
H7C	−0.0103	0.7417	0.3714	0.082*	
C8	−0.0562 (2)	0.9759 (4)	0.31701 (18)	0.0668 (12)	
H8A	−0.0638	0.9902	0.353	0.1*	
H8B	−0.1067	0.9369	0.3011	0.1*	
H8C	−0.0429	1.0699	0.3016	0.1*	
C9	0.18831 (15)	1.1593 (3)	0.40703 (9)	0.0241 (5)	
C10	0.1511 (2)	1.0770 (3)	0.45032 (11)	0.0400 (7)	
H10A	0.1924	1.0118	0.466	0.06*	
H10B	0.1043	1.0188	0.4376	0.06*	
H10C	0.1326	1.1473	0.4753	0.06*	
C11	0.12555 (16)	1.2614 (3)	0.38198 (10)	0.0314 (6)	
H11A	0.0773	1.205	0.3707	0.047*	
H11B	0.1496	1.309	0.3531	0.047*	
H11C	0.1094	1.3362	0.4061	0.047*	
C12	0.26370 (16)	1.2447 (3)	0.42515 (10)	0.0335 (6)	
H12A	0.3034	1.1776	0.4414	0.05*	
H12B	0.2478	1.3195	0.4493	0.05*	
H12C	0.2885	1.292	0.3965	0.05*	
C13	0.32921 (15)	1.1821 (3)	0.11528 (9)	0.0246 (5)	
C14	0.2878 (2)	1.2913 (3)	0.14824 (11)	0.0392 (7)	
H14A	0.3064	1.2764	0.1833	0.059*	
H14B	0.2283	1.2777	0.1451	0.059*	
H14C	0.3017	1.3909	0.1378	0.059*	
C15	0.42211 (17)	1.1921 (3)	0.12177 (12)	0.0390 (7)	
H15A	0.4474	1.1204	0.0998	0.059*	
H15B	0.4387	1.1714	0.1568	0.059*	
H15C	0.44	1.2909	0.1129	0.059*	
C16	0.30189 (18)	1.2020 (3)	0.06035 (10)	0.0350 (6)	
H16A	0.3296	1.1298	0.0398	0.052*	
H16B	0.3161	1.301	0.0494	0.052*	
H16C	0.2425	1.1882	0.0566	0.052*	
C17	0.14114 (14)	0.8571 (3)	0.05674 (9)	0.0264 (5)	
C18	0.11468 (19)	0.9139 (4)	0.00481 (12)	0.0495 (8)	
H18A	0.1394	0.8533	−0.0209	0.074*	
H18B	0.1328	1.0156	0.0013	0.074*	
H18C	0.0549	0.9092	0.0007	0.074*	
C19	0.11857 (17)	0.6972 (3)	0.06231 (12)	0.0402 (7)	
H19A	0.1432	0.6401	0.0357	0.06*	
H19B	0.0589	0.6867	0.0598	0.06*	
H19C	0.1391	0.6613	0.0951	0.06*	
C20	0.10348 (17)	0.9486 (3)	0.09720 (12)	0.0398 (7)	
H20A	0.1187	1.0516	0.0929	0.06*	
H20B	0.1236	0.9143	0.1303	0.06*	
H20C	0.0437	0.9392	0.0946	0.06*	
C21	0.41036 (15)	0.7600 (3)	0.03827 (10)	0.0269 (5)*	
C22	0.4994 (4)	0.7341 (9)	0.0418 (2)	0.0366 (16)*	0.557 (12)
H22A	0.5283	0.8282	0.0417	0.055*	0.557 (12)
H22B	0.5156	0.6754	0.0131	0.055*	0.557 (12)
H22C	0.5136	0.6816	0.073	0.055*	0.557 (12)
C23	0.3867 (4)	0.8458 (9)	−0.0118 (2)	0.0353 (15)*	0.557 (12)
H23A	0.3274	0.862	−0.014	0.053*	0.557 (12)
H23B	0.403	0.788	−0.0407	0.053*	0.557 (12)
H23C	0.4151	0.9404	−0.0116	0.053*	0.557 (12)
C24	0.3652 (4)	0.6113 (6)	0.0381 (2)	0.0345 (16)*	0.557 (12)
H24A	0.306	0.6281	0.0359	0.052*	0.557 (12)
H24B	0.3798	0.558	0.069	0.052*	0.557 (12)
H24C	0.3813	0.5535	0.0091	0.052*	0.557 (12)
C22A	0.5059 (4)	0.7874 (10)	0.0386 (3)	0.0291 (18)*	0.443 (12)
H22D	0.5302	0.7622	0.0717	0.044*	0.443 (12)
H22E	0.5165	0.8909	0.0314	0.044*	0.443 (12)
H22F	0.5302	0.7262	0.0129	0.044*	0.443 (12)
C23A	0.3726 (5)	0.7941 (11)	−0.0107 (3)	0.0321 (19)*	0.443 (12)
H23D	0.3849	0.8958	−0.0195	0.048*	0.443 (12)
H23E	0.3131	0.7814	−0.0095	0.048*	0.443 (12)
H23F	0.3942	0.7282	−0.0359	0.048*	0.443 (12)
C24A	0.3960 (5)	0.6023 (8)	0.0563 (3)	0.040 (2)*	0.443 (12)
H24D	0.4231	0.5883	0.0894	0.06*	0.443 (12)
H24E	0.4187	0.5332	0.0323	0.06*	0.443 (12)
H24F	0.3371	0.5848	0.0585	0.06*	0.443 (12)
C25	0.46391 (14)	0.9188 (3)	0.22131 (9)	0.0228 (5)	
H25	0.4551	0.8611	0.1919	0.027*	
C26	0.52586 (15)	1.0311 (3)	0.28461 (10)	0.0248 (5)	
H26	0.5669	1.0668	0.3077	0.03*	
C27	0.44361 (14)	1.0570 (2)	0.28528 (9)	0.0205 (5)	
C28	0.39708 (14)	1.1424 (3)	0.32243 (9)	0.0238 (5)	
H28A	0.3589	1.0757	0.3391	0.029*	
H28B	0.4361	1.1814	0.3485	0.029*	
Cd1	0.273167 (9)	0.943674 (18)	0.220582 (6)	0.01814 (8)	
N1	0.40537 (11)	0.9853 (2)	0.24532 (7)	0.0199 (4)	
N2	0.53725 (13)	0.9431 (2)	0.24375 (9)	0.0258 (5)	
H2	0.5844	0.9089	0.234	0.031*	
O1	0.23479 (9)	0.84275 (17)	0.30576 (6)	0.0207 (3)	
O2	0.08452 (10)	0.93091 (17)	0.33613 (6)	0.0220 (4)	
O3	0.21789 (10)	1.05130 (16)	0.37210 (6)	0.0199 (4)	
O4	0.30355 (10)	1.03704 (17)	0.13302 (6)	0.0214 (4)	
O5	0.22966 (9)	0.87083 (18)	0.05928 (6)	0.0214 (4)	
O6	0.38600 (9)	0.85444 (18)	0.07826 (6)	0.0235 (4)	
O7	0.35133 (11)	1.26197 (18)	0.30055 (7)	0.0249 (4)	
S1	0.18152 (3)	1.12091 (6)	0.25939 (2)	0.01828 (13)	
S2	0.27794 (4)	0.73133 (6)	0.16373 (2)	0.02216 (14)	
Si1	0.17558 (4)	0.97987 (7)	0.32161 (2)	0.01673 (14)	
Si2	0.29845 (4)	0.87600 (7)	0.10490 (2)	0.01808 (14)	
H7D	0.3073 (18)	1.230 (3)	0.2908 (11)	0.032 (8)*	

### Atomic displacement parameters (Å^2^)

**Table:**

	*U*^11^	*U*^22^	*U*^33^	*U*^12^	*U*^13^	*U*^23^
C1	0.0289 (13)	0.0189 (12)	0.0246 (13)	0.0058 (10)	0.0011 (10)	0.0068 (10)
C2	0.0374 (14)	0.0285 (14)	0.0228 (13)	0.0034 (11)	0.0033 (11)	0.0067 (11)
C3	0.0305 (15)	0.0423 (17)	0.0537 (19)	0.0115 (13)	0.0052 (13)	0.0252 (15)
C4	0.070 (2)	0.0199 (13)	0.0316 (16)	0.0063 (13)	0.0060 (14)	0.0035 (12)
C5	0.0174 (11)	0.0277 (13)	0.0262 (13)	−0.0069 (10)	0.0006 (9)	0.0011 (10)
C6	0.0413 (18)	0.104 (3)	0.0351 (18)	−0.039 (2)	0.0027 (14)	−0.0169 (19)
C7	0.0508 (19)	0.049 (2)	0.061 (2)	−0.0304 (16)	−0.0249 (16)	0.0225 (17)
C8	0.0348 (18)	0.055 (2)	0.108 (3)	0.0125 (16)	−0.025 (2)	−0.025 (2)
C9	0.0268 (13)	0.0268 (13)	0.0190 (12)	−0.0028 (10)	0.0055 (10)	−0.0072 (10)
C10	0.0499 (18)	0.0429 (17)	0.0283 (15)	−0.0050 (14)	0.0163 (13)	0.0008 (13)
C11	0.0331 (14)	0.0291 (14)	0.0319 (15)	0.0023 (11)	0.0015 (11)	−0.0098 (11)
C12	0.0352 (15)	0.0379 (15)	0.0272 (14)	−0.0035 (12)	0.0009 (11)	−0.0117 (12)
C13	0.0366 (14)	0.0151 (11)	0.0223 (13)	−0.0049 (10)	0.0042 (10)	0.0033 (10)
C14	0.0596 (19)	0.0186 (13)	0.0402 (17)	−0.0046 (13)	0.0145 (14)	−0.0015 (12)
C15	0.0404 (16)	0.0299 (15)	0.0466 (18)	−0.0127 (12)	0.0011 (13)	0.0064 (13)
C16	0.0544 (18)	0.0249 (14)	0.0254 (14)	−0.0051 (12)	−0.0007 (12)	0.0069 (11)
C17	0.0173 (12)	0.0373 (15)	0.0247 (13)	0.0020 (10)	0.0023 (9)	−0.0056 (11)
C18	0.0286 (15)	0.083 (2)	0.0361 (17)	0.0086 (16)	−0.0062 (13)	0.0082 (17)
C19	0.0319 (15)	0.0428 (17)	0.0459 (18)	−0.0100 (13)	0.0021 (13)	−0.0115 (14)
C20	0.0256 (14)	0.0496 (19)	0.0447 (18)	0.0052 (12)	0.0082 (12)	−0.0174 (14)
C25	0.0219 (12)	0.0215 (12)	0.0253 (13)	0.0008 (9)	0.0036 (10)	−0.0024 (10)
C26	0.0195 (12)	0.0224 (12)	0.0324 (14)	−0.0008 (9)	−0.0011 (10)	−0.0014 (10)
C27	0.0188 (12)	0.0198 (12)	0.0229 (13)	−0.0020 (9)	0.0007 (9)	0.0036 (9)
C28	0.0199 (12)	0.0270 (13)	0.0243 (13)	−0.0017 (10)	0.0001 (9)	−0.0031 (10)
Cd1	0.01793 (11)	0.01975 (12)	0.01684 (11)	0.00073 (6)	0.00194 (7)	−0.00169 (6)
N1	0.0179 (10)	0.0200 (10)	0.0217 (10)	0.0015 (8)	0.0020 (8)	0.0005 (8)
N2	0.0174 (10)	0.0237 (11)	0.0366 (13)	0.0030 (8)	0.0048 (9)	0.0001 (9)
O1	0.0246 (8)	0.0175 (8)	0.0202 (8)	0.0042 (7)	0.0029 (6)	0.0038 (7)
O2	0.0192 (8)	0.0248 (9)	0.0223 (9)	−0.0067 (6)	0.0031 (7)	−0.0003 (7)
O3	0.0196 (8)	0.0216 (9)	0.0186 (8)	−0.0008 (6)	0.0004 (7)	−0.0019 (6)
O4	0.0298 (9)	0.0154 (8)	0.0192 (9)	−0.0034 (7)	0.0046 (7)	0.0011 (6)
O5	0.0194 (8)	0.0263 (9)	0.0186 (8)	−0.0003 (7)	0.0013 (6)	−0.0020 (7)
O6	0.0215 (8)	0.0284 (9)	0.0207 (9)	−0.0007 (7)	0.0028 (7)	−0.0048 (7)
O7	0.0186 (9)	0.0203 (9)	0.0361 (10)	−0.0023 (7)	0.0049 (7)	−0.0036 (7)
S1	0.0180 (3)	0.0178 (3)	0.0192 (3)	0.0024 (2)	0.0022 (2)	0.0024 (2)
S2	0.0313 (3)	0.0164 (3)	0.0190 (3)	−0.0008 (2)	0.0029 (2)	0.0002 (2)
Si1	0.0153 (3)	0.0172 (3)	0.0179 (3)	−0.0018 (2)	0.0027 (2)	−0.0009 (2)
Si2	0.0209 (3)	0.0174 (3)	0.0160 (3)	−0.0005 (2)	0.0023 (2)	−0.0008 (2)

### Geometric parameters (Å, °)

**Table:**

C1—O1	1.466 (3)	C18—H18C	0.98
C1—C4	1.517 (4)	C19—H19A	0.98
C1—C3	1.521 (4)	C19—H19B	0.98
C1—C2	1.522 (3)	C19—H19C	0.98
C2—H2A	0.98	C20—H20A	0.98
C2—H2B	0.98	C20—H20B	0.98
C2—H2C	0.98	C20—H20C	0.98
C3—H3A	0.98	C21—O6	1.439 (3)
C3—H3B	0.98	C21—C23A	1.454 (7)
C3—H3C	0.98	C21—C22	1.473 (6)
C4—H4A	0.98	C21—C24A	1.539 (8)
C4—H4B	0.98	C21—C24	1.545 (6)
C4—H4C	0.98	C21—C22A	1.580 (7)
C5—O2	1.455 (3)	C21—C23	1.581 (7)
C5—C8	1.499 (4)	C22—H22A	0.98
C5—C6	1.505 (4)	C22—H22B	0.98
C5—C7	1.513 (4)	C22—H22C	0.98
C6—H6A	0.98	C23—H23A	0.98
C6—H6B	0.98	C23—H23B	0.98
C6—H6C	0.98	C23—H23C	0.98
C7—H7A	0.98	C24—H24A	0.98
C7—H7B	0.98	C24—H24B	0.98
C7—H7C	0.98	C24—H24C	0.98
C8—H8A	0.98	C22A—H22D	0.98
C8—H8B	0.98	C22A—H22E	0.98
C8—H8C	0.98	C22A—H22F	0.98
C9—O3	1.452 (3)	C23A—H23D	0.98
C9—C12	1.520 (3)	C23A—H23E	0.98
C9—C11	1.521 (4)	C23A—H23F	0.98
C9—C10	1.523 (4)	C24A—H24D	0.98
C10—H10A	0.98	C24A—H24E	0.98
C10—H10B	0.98	C24A—H24F	0.98
C10—H10C	0.98	C25—N1	1.319 (3)
C11—H11A	0.98	C25—N2	1.336 (3)
C11—H11B	0.98	C25—H25	0.95
C11—H11C	0.98	C26—C27	1.365 (3)
C12—H12A	0.98	C26—N2	1.372 (3)
C12—H12B	0.98	C26—H26	0.95
C12—H12C	0.98	C27—N1	1.378 (3)
C13—O4	1.472 (3)	C27—C28	1.492 (3)
C13—C14	1.507 (4)	C28—O7	1.433 (3)
C13—C15	1.523 (4)	C28—H28A	0.99
C13—C16	1.525 (3)	C28—H28B	0.99
C14—H14A	0.98	Cd1—N1	2.2653 (19)
C14—H14B	0.98	Cd1—S1	2.4599 (6)
C14—H14C	0.98	Cd1—S2	2.4633 (6)
C15—H15A	0.98	Cd1—O1	2.5511 (16)
C15—H15B	0.98	Cd1—O4	2.5516 (16)
C15—H15C	0.98	N2—H2	0.88
C16—H16A	0.98	O1—Si1	1.6471 (16)
C16—H16B	0.98	O2—Si1	1.6147 (17)
C16—H16C	0.98	O3—Si1	1.6250 (17)
C17—O5	1.450 (3)	O4—Si2	1.6505 (17)
C17—C20	1.514 (4)	O5—Si2	1.6236 (16)
C17—C19	1.514 (4)	O6—Si2	1.6335 (17)
C17—C18	1.524 (4)	O7—H7D	0.81 (3)
C18—H18A	0.98	S1—Si1	2.1047 (8)
C18—H18B	0.98	S2—Si2	2.0872 (8)
			
O1—C1—C4	106.44 (19)	C17—C20—H20B	109.5
O1—C1—C3	106.70 (19)	H20A—C20—H20B	109.5
C4—C1—C3	110.8 (2)	C17—C20—H20C	109.5
O1—C1—C2	111.21 (19)	H20A—C20—H20C	109.5
C4—C1—C2	110.8 (2)	H20B—C20—H20C	109.5
C3—C1—C2	110.7 (2)	O6—C21—C23A	114.6 (4)
C1—C2—H2A	109.5	O6—C21—C22	110.5 (3)
C1—C2—H2B	109.5	C23A—C21—C22	118.4 (4)
H2A—C2—H2B	109.5	O6—C21—C24A	106.2 (3)
C1—C2—H2C	109.5	C23A—C21—C24A	114.5 (5)
H2A—C2—H2C	109.5	C22—C21—C24A	89.6 (4)
H2B—C2—H2C	109.5	O6—C21—C24	112.5 (3)
C1—C3—H3A	109.5	C23A—C21—C24	90.0 (4)
C1—C3—H3B	109.5	C22—C21—C24	109.2 (4)
H3A—C3—H3B	109.5	O6—C21—C22A	101.7 (3)
C1—C3—H3C	109.5	C23A—C21—C22A	110.8 (4)
H3A—C3—H3C	109.5	C24A—C21—C22A	108.0 (4)
H3B—C3—H3C	109.5	C24—C21—C22A	127.6 (4)
C1—C4—H4A	109.5	O6—C21—C23	105.3 (3)
C1—C4—H4B	109.5	C22—C21—C23	110.0 (4)
H4A—C4—H4B	109.5	C24A—C21—C23	133.7 (4)
C1—C4—H4C	109.5	C24—C21—C23	109.4 (4)
H4A—C4—H4C	109.5	C22A—C21—C23	97.7 (4)
H4B—C4—H4C	109.5	C21—C22—H22A	109.5
O2—C5—C8	106.7 (2)	C21—C22—H22B	109.5
O2—C5—C6	112.2 (2)	H22A—C22—H22B	109.5
C8—C5—C6	110.6 (3)	C21—C22—H22C	109.5
O2—C5—C7	106.1 (2)	H22A—C22—H22C	109.5
C8—C5—C7	110.8 (3)	H22B—C22—H22C	109.5
C6—C5—C7	110.3 (3)	C21—C23—H23A	109.5
C5—C6—H6A	109.5	C21—C23—H23B	109.5
C5—C6—H6B	109.5	H23A—C23—H23B	109.5
H6A—C6—H6B	109.5	C21—C23—H23C	109.5
C5—C6—H6C	109.5	H23A—C23—H23C	109.5
H6A—C6—H6C	109.5	H23B—C23—H23C	109.5
H6B—C6—H6C	109.5	C21—C24—H24A	109.5
C5—C7—H7A	109.5	C21—C24—H24B	109.5
C5—C7—H7B	109.5	H24A—C24—H24B	109.5
H7A—C7—H7B	109.5	C21—C24—H24C	109.5
C5—C7—H7C	109.5	H24A—C24—H24C	109.5
H7A—C7—H7C	109.5	H24B—C24—H24C	109.5
H7B—C7—H7C	109.5	C21—C22A—H22D	109.5
C5—C8—H8A	109.5	C21—C22A—H22E	109.5
C5—C8—H8B	109.5	H22D—C22A—H22E	109.5
H8A—C8—H8B	109.5	C21—C22A—H22F	109.5
C5—C8—H8C	109.5	H22D—C22A—H22F	109.5
H8A—C8—H8C	109.5	H22E—C22A—H22F	109.5
H8B—C8—H8C	109.5	C21—C23A—H23D	109.5
O3—C9—C12	105.42 (19)	C21—C23A—H23E	109.5
O3—C9—C11	111.74 (19)	H23D—C23A—H23E	109.5
C12—C9—C11	110.4 (2)	C21—C23A—H23F	109.5
O3—C9—C10	107.7 (2)	H23D—C23A—H23F	109.5
C12—C9—C10	110.8 (2)	H23E—C23A—H23F	109.5
C11—C9—C10	110.7 (2)	C21—C24A—H24D	109.5
C9—C10—H10A	109.5	C21—C24A—H24E	109.5
C9—C10—H10B	109.5	H24D—C24A—H24E	109.5
H10A—C10—H10B	109.5	C21—C24A—H24F	109.5
C9—C10—H10C	109.5	H24D—C24A—H24F	109.5
H10A—C10—H10C	109.5	H24E—C24A—H24F	109.5
H10B—C10—H10C	109.5	N1—C25—N2	111.1 (2)
C9—C11—H11A	109.5	N1—C25—H25	124.4
C9—C11—H11B	109.5	N2—C25—H25	124.4
H11A—C11—H11B	109.5	C27—C26—N2	106.0 (2)
C9—C11—H11C	109.5	C27—C26—H26	127
H11A—C11—H11C	109.5	N2—C26—H26	127
H11B—C11—H11C	109.5	C26—C27—N1	108.9 (2)
C9—C12—H12A	109.5	C26—C27—C28	128.8 (2)
C9—C12—H12B	109.5	N1—C27—C28	122.3 (2)
H12A—C12—H12B	109.5	O7—C28—C27	113.51 (19)
C9—C12—H12C	109.5	O7—C28—H28A	108.9
H12A—C12—H12C	109.5	C27—C28—H28A	108.9
H12B—C12—H12C	109.5	O7—C28—H28B	108.9
O4—C13—C14	105.52 (19)	C27—C28—H28B	108.9
O4—C13—C15	108.2 (2)	H28A—C28—H28B	107.7
C14—C13—C15	111.2 (2)	N1—Cd1—S1	110.88 (5)
O4—C13—C16	109.86 (19)	N1—Cd1—S2	104.96 (5)
C14—C13—C16	111.1 (2)	S1—Cd1—S2	144.16 (2)
C15—C13—C16	110.8 (2)	N1—Cd1—O1	93.81 (6)
C13—C14—H14A	109.5	S1—Cd1—O1	71.84 (4)
C13—C14—H14B	109.5	S2—Cd1—O1	106.15 (4)
H14A—C14—H14B	109.5	N1—Cd1—O4	89.58 (6)
C13—C14—H14C	109.5	S1—Cd1—O4	107.97 (4)
H14A—C14—H14C	109.5	S2—Cd1—O4	71.83 (4)
H14B—C14—H14C	109.5	O1—Cd1—O4	176.43 (5)
C13—C15—H15A	109.5	C25—N1—C27	106.15 (19)
C13—C15—H15B	109.5	C25—N1—Cd1	118.84 (16)
H15A—C15—H15B	109.5	C27—N1—Cd1	134.62 (16)
C13—C15—H15C	109.5	C25—N2—C26	107.8 (2)
H15A—C15—H15C	109.5	C25—N2—H2	126.1
H15B—C15—H15C	109.5	C26—N2—H2	126.1
C13—C16—H16A	109.5	C1—O1—Si1	133.23 (14)
C13—C16—H16B	109.5	C1—O1—Cd1	129.74 (13)
H16A—C16—H16B	109.5	Si1—O1—Cd1	96.97 (7)
C13—C16—H16C	109.5	C5—O2—Si1	136.55 (15)
H16A—C16—H16C	109.5	C9—O3—Si1	131.46 (15)
H16B—C16—H16C	109.5	C13—O4—Si2	131.67 (15)
O5—C17—C20	110.6 (2)	C13—O4—Cd1	131.47 (13)
O5—C17—C19	109.0 (2)	Si2—O4—Cd1	96.30 (7)
C20—C17—C19	110.8 (2)	C17—O5—Si2	134.18 (14)
O5—C17—C18	105.0 (2)	C21—O6—Si2	131.80 (15)
C20—C17—C18	110.5 (2)	C28—O7—H7D	108 (2)
C19—C17—C18	110.8 (2)	Si1—S1—Cd1	88.67 (3)
C17—C18—H18A	109.5	Si2—S2—Cd1	88.52 (3)
C17—C18—H18B	109.5	O2—Si1—O3	106.16 (9)
H18A—C18—H18B	109.5	O2—Si1—O1	114.02 (9)
C17—C18—H18C	109.5	O3—Si1—O1	106.40 (9)
H18A—C18—H18C	109.5	O2—Si1—S1	115.45 (7)
H18B—C18—H18C	109.5	O3—Si1—S1	112.07 (7)
C17—C19—H19A	109.5	O1—Si1—S1	102.53 (6)
C17—C19—H19B	109.5	O5—Si2—O6	105.25 (8)
H19A—C19—H19B	109.5	O5—Si2—O4	112.77 (9)
C17—C19—H19C	109.5	O6—Si2—O4	105.98 (9)
H19A—C19—H19C	109.5	O5—Si2—S2	114.67 (7)
H19B—C19—H19C	109.5	O6—Si2—S2	114.80 (7)
C17—C20—H20A	109.5	O4—Si2—S2	103.24 (7)
			
N2—C26—C27—N1	0.1 (3)	S2—Cd1—O4—Si2	2.44 (6)
N2—C26—C27—C28	178.1 (2)	C20—C17—O5—Si2	41.1 (3)
C26—C27—C28—O7	121.6 (3)	C19—C17—O5—Si2	−80.9 (3)
N1—C27—C28—O7	−60.7 (3)	C18—C17—O5—Si2	160.4 (2)
N2—C25—N1—C27	0.3 (3)	C23A—C21—O6—Si2	67.3 (5)
N2—C25—N1—Cd1	−173.57 (15)	C22—C21—O6—Si2	−155.9 (4)
C26—C27—N1—C25	−0.3 (3)	C24A—C21—O6—Si2	−60.2 (4)
C28—C27—N1—C25	−178.4 (2)	C24—C21—O6—Si2	−33.6 (4)
C26—C27—N1—Cd1	172.21 (17)	C22A—C21—O6—Si2	−173.1 (4)
C28—C27—N1—Cd1	−5.9 (3)	C23—C21—O6—Si2	85.4 (4)
S1—Cd1—N1—C25	−167.65 (16)	N1—Cd1—S1—Si1	−87.21 (6)
S2—Cd1—N1—C25	12.51 (19)	S2—Cd1—S1—Si1	92.52 (4)
O1—Cd1—N1—C25	120.35 (18)	O1—Cd1—S1—Si1	−0.09 (4)
O4—Cd1—N1—C25	−58.54 (18)	O4—Cd1—S1—Si1	176.16 (4)
S1—Cd1—N1—C27	20.6 (2)	N1—Cd1—S2—Si2	−86.42 (6)
S2—Cd1—N1—C27	−159.2 (2)	S1—Cd1—S2—Si2	93.83 (4)
O1—Cd1—N1—C27	−51.4 (2)	O1—Cd1—S2—Si2	175.01 (4)
O4—Cd1—N1—C27	129.7 (2)	O4—Cd1—S2—Si2	−1.92 (4)
N1—C25—N2—C26	−0.3 (3)	C5—O2—Si1—O3	175.4 (2)
C27—C26—N2—C25	0.1 (3)	C5—O2—Si1—O1	−67.8 (2)
C4—C1—O1—Si1	124.2 (2)	C5—O2—Si1—S1	50.6 (2)
C3—C1—O1—Si1	−117.5 (2)	C9—O3—Si1—O2	−47.8 (2)
C2—C1—O1—Si1	3.4 (3)	C9—O3—Si1—O1	−169.64 (18)
C4—C1—O1—Cd1	−59.4 (2)	C9—O3—Si1—S1	79.05 (19)
C3—C1—O1—Cd1	59.0 (3)	C1—O1—Si1—O2	−57.3 (2)
C2—C1—O1—Cd1	179.85 (14)	Cd1—O1—Si1—O2	125.37 (8)
N1—Cd1—O1—C1	−66.57 (18)	C1—O1—Si1—O3	59.3 (2)
S1—Cd1—O1—C1	−177.30 (18)	Cd1—O1—Si1—O3	−117.96 (8)
S2—Cd1—O1—C1	40.21 (17)	C1—O1—Si1—S1	177.14 (18)
N1—Cd1—O1—Si1	110.85 (8)	Cd1—O1—Si1—S1	−0.14 (6)
S1—Cd1—O1—Si1	0.12 (5)	Cd1—S1—Si1—O2	−124.43 (7)
S2—Cd1—O1—Si1	−142.37 (6)	Cd1—S1—Si1—O3	113.86 (7)
C8—C5—O2—Si1	−121.4 (3)	Cd1—S1—Si1—O1	0.14 (6)
C6—C5—O2—Si1	−0.2 (4)	C17—O5—Si2—O6	167.1 (2)
C7—C5—O2—Si1	120.3 (3)	C17—O5—Si2—O4	−77.9 (2)
C12—C9—O3—Si1	−146.96 (18)	C17—O5—Si2—S2	39.9 (2)
C11—C9—O3—Si1	−27.0 (3)	C21—O6—Si2—O5	−40.8 (2)
C10—C9—O3—Si1	94.7 (2)	C21—O6—Si2—O4	−160.5 (2)
C14—C13—O4—Si2	156.53 (19)	C21—O6—Si2—S2	86.2 (2)
C15—C13—O4—Si2	−84.3 (3)	C13—O4—Si2—O5	−66.5 (2)
C16—C13—O4—Si2	36.7 (3)	Cd1—O4—Si2—O5	121.51 (8)
C14—C13—O4—Cd1	−34.2 (3)	C13—O4—Si2—O6	48.1 (2)
C15—C13—O4—Cd1	85.0 (2)	Cd1—O4—Si2—O6	−123.85 (8)
C16—C13—O4—Cd1	−153.95 (17)	C13—O4—Si2—S2	169.15 (19)
N1—Cd1—O4—C13	−63.63 (19)	Cd1—O4—Si2—S2	−2.81 (7)
S1—Cd1—O4—C13	48.22 (19)	Cd1—S2—Si2—O5	−120.17 (7)
S2—Cd1—O4—C13	−169.54 (19)	Cd1—S2—Si2—O6	117.74 (7)
N1—Cd1—O4—Si2	108.35 (8)	Cd1—S2—Si2—O4	2.90 (7)
S1—Cd1—O4—Si2	−139.79 (6)		

### Hydrogen-bond geometry (Å, °)

**Table:**

*D*—H···*A*	*D*—H	H···*A*	*D*···*A*	*D*—H···*A*
N2—H2···O7^i^	0.88	1.96	2.759 (3)	151
O7—H7D···S1	0.81 (3)	2.41 (3)	3.2119 (19)	176 (3)

Symmetry codes: (i) −*x*+1, *y*−1/2, −*z*+1/2.
